# Transmission of Human Papillomavirus in Heterosexual Couples

**DOI:** 10.3201/eid1406.070616.2

**Published:** 2008-06

**Authors:** Brenda Y. Hernandez, Lynne R. Wilkens, Xuemei Zhu, Pamela Thompson, Katharine McDuffie, Yurii B. Shvetsov, Lori E. Kamemoto, Jeffrey Killeen, Lily Ning, Marc T. Goodman

**Affiliations:** * University of Hawaii, Manoa, Hawaii, USA; † University of Hawaii John A. Burns School of Medicine, Honolulu, Hawaii, USA; ‡ Kapi'olani Medical Center for Women and Children, Honolulu, Hawaii, USA

**Keywords:** Human papillomavirus (HPV), transmission, couples, partners, research

## Abstract

We examined the transmission of human papillomavirus (HPV) in 25 heterosexual, monogamous couples (25 men, 25 women), followed up over an average of 7.5 months. A total of 53 heterosexual transmission events were observed among 16 couples (14 male-to-female and 39 female-to-male). Sexual transmission involved 13 different oncogenic and nononcogenic HPV types; 8% were vaccine-covered types transmitted between partners. The overall rate of HPV transmission from the penis to the cervix was 4.9/100 person-months, which was substantially lower than that from the cervix to the penis (17.4/100 person-months). Transmission between the hands and genitals, as well as apparent self-inoculation events (primarily in men), were also observed. Couples who transmitted HPV were more sexually active and used condoms less frequently. These results have implications for HPV prevention and control strategies, including the targeting of prophylactic vaccines.

## Abstract 2

Rate of transmission from penis to cervix was lower than that from cervix to penis; 13 different genotypes were sexually transmitted.

Cervical cancer remains a major source of illness and death among women globally, and infection with oncogenic human papillomaviruses (HPVs) is its principal cause (*1*,*2*). Men are assumed to be the main reservoirs of genital HPV infection for women, although comparatively little is known about the natural history of HPV in men.

A limited number of cross-sectional and case-control studies have evaluated genotype-specific HPV concordance in male-female couples (*3*–*7*). There are, however, no empirical data on the heterosexual transmission of HPV. Our investigation evaluates the transmission of HPV in a cohort of male-female sexual partners.

## Methods

### Study Participants

The study was conducted at the University Health Services of the University of Hawaii at Manoa from February 2005 through November 2006. Promotional efforts included flyers and email invitations, which were part of larger ongoing studies of HPV. The study was approved by the Committee on Human Studies of the University of Hawaii. All study participants provided written informed consent. Eligible participants were at least 18 years of age, English-speaking, not currently pregnant, and in a monogamous relationship with the index partner.

### Specimen Collection

Couples attended concurrent study visits at 2-month intervals. Trained clinicians collected exfoliated cell samples for HPV DNA detection. For men, separate genital specimens from the penis glans/corona, penis shaft, scrotum, and inner foreskin (uncircumcised men) were collected by using textured paper and a saline-moistened swab (*8*,*9*). Anal canal specimens were collected by using a saline-moistened swab. A cytobrush was used to collect oral specimens (buccal cavity, tongue). Specimens from the dominant hand (palm, fingertips, under the fingernails) were collected by using a saline-moistened swab. Participants self-collected first-catch urine samples (30 mL) at the clinic. Using latex gloves, men collected semen specimens at home during masturbation within 24 hours of each visit.

For women, a cervical cytology (Papanicolaou [Pap]) smear was collected, and a swab and cytobrush were used to consecutively sample the ectocervix and endocervix, including the transformation zone. The same methods used for collection of anal, oral, hand, and urine specimens from men were used for collection of specimens from women.

### HPV DNA Testing and Genotyping

DNA was extracted from specimens by using commercial reagents (QIAGEN, Valencia, CA, USA). The PCR used PGMY09/PGMY11 primers to amplify a 450-bp region of the L1 HPV genome (*10*). Amplification of the human β-globin gene was included as an internal control for sample sufficiency. HPV-positive specimens were subsequently genotyped by using commercial reagents (Roche Molecular Systems Inc., Branchburg, NJ, USA) originating from a prototype line blot assay (*11*). The assay detects 37 different HPV types, including oncogenic/probable oncogenic types (HPV 16, 18, 26, 31, 33, 35, 39, 45, 51, 52, 53, 56, 58, 59, 66, 68, 73, 82, and IS39, a subtype of HPV 82), nononcogenic types (HPV 6, 11, 40, 42, 54, 61, 70, 72, 81, and CP6108, also known as candidate HPV 89), and types with undetermined risk status (HPV 55, 62, 64, 67, 69, 71, 83, and 84) (*12*,*13*). This PCR-based assay has demonstrated a high degree of sensitivity and reproducibility (*14*–*16*). HPV testing and genotyping procedures have been given in detail previously (*9*).

### Statistical Analysis

The main objective of the statistical analysis was to evaluate HPV transmission between partners. Each type-specific HPV infection was assigned a status of transmitted or not transmitted by time period and anatomic site. For some statistics, the penis anatomic site was further divided into foreskin, glans, shaft, and urine, which is a proxy for urethral infection. HPV in female urine, which has demonstrated type-specific concordance with cervical measurements (*17*), was considered to be a proxy for cervical and other lower genital tract infections. Therefore, cervix and urine were combined as 1 anatomic site. When there were >1 possible source sites, sites were grouped and evaluated as 1 transmission event. When there were >2 destination sites, each was counted as a separate transmission event.

Partner transmission was defined as the presence of a specific HPV genotype at an anatomic site in 1 partner and its absence in all sites of the other partner at a given visit, along with the presence of this HPV type in the unaffected partner at the subsequent visit. Auto-inoculation was defined as the presence of a particular HPV type at an anatomic site in 1 partner and its absence in all sites of the other partner at a given visit, and the presence of this HPV type in a different anatomic site in the affected partner at the subsequent visit. An event was defined as self-inoculation only after possible transmission from the partner was ruled out, i.e., when the partner was negative for the HPV genotype at prior and concurrent visits.

The rate of HPV transmission was calculated as the number of HPV type-specific transmission events divided by the number of person-months of exposure × 100 and expressed as the rate per 100 person-months. Exact confidence intervals (CIs) for transmission rates were calculated by assuming a Poisson distribution for the number of events (*18*). Person-months of exposure for each HPV infection by anatomic site were computed based on the period of time between successive visits. When HPV was detected at a given visit and HPV type was absent at the successive visit, the exposure period was estimated at half of the visit interval. Comparisons between couples by transmission status were made by using the *t* test, χ^2^ statistic, and linear rank statistic.

## Results

### Study Participants

Thirty-eight couples (38 men, 38 women) were enrolled. Six couples left the study, including 2 whose relationships ended. The present analysis focuses on 25 couples with at least 2 visits. Couples were followed up at ≈2-month intervals over an average of 7.5 months.

The mean age was 28 years (range 18–59 years) for men and 26 years (range 18–57 years) for women. Participants comprised Caucasians (52%), Asians (8%), Pacific Islanders (6%), and persons of other single- or mixed-race backgrounds (34%). Couples were single/never married (48%), living together as married (32%), separated/divorced/widowed (12%), and married (8%). All participants indicated that the index partner was their only current sexual partner, and all reported vaginal intercourse with penile penetration during the follow-up period.

Five men were uncircumcised. At study entry, 1 woman had cervical low-grade squamous intraepithelial lesions, and 2 men had genital warts. Cervical atypia was diagnosed in 2 women during follow-up.

### HPV Transmission

Overall, 957 (94%) of 1,015 specimens were adequate based on β-globin detection and were included in the present analyses. At study entry, 7 couples were HPV negative at all anatomic sites, 4 couples had 1 HPV-positive partner (3 were anal infections in the man), and 14 couples had both partners HPV positive. Of the 14 couples positive for HPV, 3 couples were positive for different HPV types, and 11 were positive for the same HPV type(s) at 1 or more anatomic sites (Table 1). All 11 HPV type-specific concordant infections involved the penis in the male partner and, with 2 exceptions, the cervix and/or urine in the female partner.

**Table 1:** HPV status of couples at study entry*

HPV status at baseline	No.	Man			Woman	
		Site	HPV		Site	HPV
Both partners HPV neg	7	All	Neg		All	Neg
One partner HPV pos	4	Anus	18		All	Neg
		Anus	18		All	Neg
		Anus	42/51/52/84		All	Neg
		All	Neg		Urine	CP6108
Both partners HPV pos						
Different HPV type(s)	3	Anus	6		Anus	39
		Shaft	6		Cervix/urine	16
		Shaft, scrotum	6		Cervix/urine, anus	31
One or more of the same HPV type(s)†	11	Shaft	59		Cervix	59
		Shaft	59		Hand	59
		Shaft	84		Cervix	84
		Shaft	62		Urine	62
		Shaft	55		Anus	55
		Glans, shaft	56, 59		Cervix/urine, anus, hand	56, 59
		Shaft, scrotum	62		Urine, anus, hand	62
		Foreskin , glans, shaft, scrotum	CP6108		Urine, anus	CP6108
		Shaft, scrotum	42		Cervix/urine, anus	42
		Glans, shaft, scrotum	53		Cervix	53

*HPV, human papillomavirus; neg, negative; pos, positive. †Only concordant types shown.

All transmission events occurred in couples with 1 or both partners positive for HPV at study entry. A total of 78 transmission events were observed in 16 couples, including 14 male-to-female, 39 female-to-male, 21 male auto-inoculation, and 4 female self-inoculation (Appendix Table). Overall, 15 different genotypes were transmitted, including oncogenic types and nononcogenic/undetermined risk types. Partner transmission involved 13 genotypes. Forty-one (53%) of the 78 transmission events involved multiple potential sources of infection.

Male-to-female transmission was observed in 7 couples. All infections transmitted from male to female partners originated in the penis with or without additional involvement of the scrotum. In particular, the penis shaft was a predominant source of infection either alone or with other genital sites. The cervix and anus were the most frequent targets of transmission from the men. Transmission of oncogenic HPV 16 comprised 1 of the 5 genital-to-cervix events. Male genital to female hand transmission was observed in 3 couples (D, E, and G).

Female-to-male transmission was observed in 12 couples. Transmission from the cervix and/or urine to the male genitals comprised most female-to-male events. Within penis subsites, the glans was targeted more frequently than the shaft. The anus was frequently an additional source site along with the cervix/urine. In addition, the anus was the sole source of transmission to the male genitals on 4 occasions, of which 3 targeted the partner’s scrotum (couples A, B, and J). The penis and scrotum, respectively, were the most frequent targets of infection from women. There were 4 instances of transmission from the woman’s hands to the man’s genitals, including 1 case in which it was the sole source (couple I). Oncogenic HPV types 16 and 18 were transmitted in only 2 of the 39 female-to-male events.

Male self-inoculation was observed in 11 men, including 3 for whom no heterosexual transmission was observed during the entire period of follow-up. Sixteen events involved transmission between different genital sites, 2 involved anal-to-genital transmission, and 3 involved genital-to-hand transmission. Most genital-to-genital events involved transmission between penis subsites. In 3 instances, male self-inoculation immediately preceded transmission of HPV to female partners (couples A and B).

Female self-inoculation was observed in 4 females. All involved urine as a source site and 3 of these targeted the hands.

Heterosexual transmission of the same HPV genotype to >1 anatomic site (excluding penis subsites) was observed on 13 occasions. Eight of these cases involved transmission from the cervix/urine to the penis and scrotum.

Heterosexual transmission of multiple genotypes to the same anatomic site during the same period was observed in 5 instances (couples A, B, D, F, and G); the scrotum was the target site on 3 of these occasions. In 1 case, different genotypes in the cervix and urine were each transmitted to both the penis glans and shaft (couple K).

Transmission of some HPV types in preference to others was also observed. In couple L, after cervical infection with HPV 16 and 31 was detected at study entry, only HPV 31 was transmitted to the penis (glans, shaft) and scrotum. Alternatively, the female anus may have been the source of infection because HPV 31, not HPV 16, was present in the anus at baseline. In couple D, HPV 16, 59, and 62 were present in the penis shaft at baseline. Subsequently, only HPV 16 was transmitted to the woman’s cervix/urine and anus by the second visit.

A number of instances indicated apparent reinfection after viral clearance. In 1 couple (A), the woman’s anus was positive for HPV 39 infection at baseline and at visit 2. By visit 3, HPV 39 had been transmitted to the man’s scrotum, and the woman was negative for HPV 39. HPV 39 had been transmitted (presumably by auto-inoculation) to the penis by visit 4, and the infection remained through visit 5. By visit 6, or 9 months after initial clearance in the woman, HPV 39 had been transmitted to the woman’s anus.

Four cases of transmission to the female anus required >4 months of exposure to an infected partner. In 27 incident infections, no source of infection could be ascertained; 17 of these infections were in the male genitals.

The rates of HPV transmission by source site are shown in Table 2. Overall, the rate of transmission from the penis to the cervix/urine was 4.9 per 100 person-months of exposure (95% CI 1.6–10.0). By contrast, the overall rate of transmission from the cervix/urine to the penis was 17.4 per 100 person-months of exposure (95% CI 10.6–25.8). Transmission of oncogenic types to the male genitals was greater from the female urine alone than from the cervix alone. Transmission of HPV from the penis to the female anus was higher than that to the cervix; this was particularly true for transmission of oncogenic types to the anus (12.2 per 100 person-months of exposure, 95% CI 3.9–24.9). The highest rates of transmission were observed from the female anus to the male genitals (47.1 per 100 person-months of exposure, 95% CI 30.2–67.7), followed by cervix to the male genitals (27.8 per 100 person-months of exposure, 95% CI 19.0–38.3). In men, the rate of transmission by auto-inoculation was comparable to that of transmission from women. For example, the rate of transmission from the scrotum alone to the penis (6.0 per 100 person-months of exposure, 95% CI 1.2–14.5) was comparable to that of cervix only to penis (5.0 per 100 person-months of exposure, 95% CI 1.0–11.9).

**Table 2:** Rate of HPV transmission by source and target site in male-female couples*

Source site	Target site	No. transmission events			Duration of exposure, mo†			Transmission rate/100 person-months (95% CI)	
		Overall	Oncogenic		Overall	Oncogenic		Overall	Oncogenic‡
Male to female									
Penis only	Cervix/urine	2	1		102	41		1.9 (0.2–5.4)	2.4 (0.06–9.0)
Penis only	Anus	3	3		102	41		2.9 (0.6–7.1)	7.3 (1.5–17.6)
Penis only	Hand	2	1		102	41		1.9 (0.2–5.4)	2.4 (0.06–9.0)
Any penis	Cervix/urine	5	1		102	41		4.9 (1.6–10.0)	2.4 (0.06–9.0)
Any penis	Anus	6	5		102	41		5.9 (2.2–11.4)	12.2 (3.9–24.9)
Any penis	Hand	3	2		102	41		2.9 (0.6–7.1)	4.9 (0.6–13.5)
Any scrotum	Cervix/urine	3	0		50	–		6.0 (1.2–14.5)	–
Any scrotum	Anus	3	2		50	27		6.0 (1.2–14.5)	7.5 (0.9–20.9)
Any genital	Cervix/urine	5	1		111	51		4.5 (1.5–9.3)	2.0 (0.1–7.2)
Any genital	Anus	6	5		111	51		5.4 (2.0–10.6)	9.8 (3.2–20.0)
Female to male									
Cervix only	Penis	3	0		61	–		5.0 (1.0–11.9)	–
Cervix only	Scrotum	1	0		61	–		1.6 (0.04–6.1)	–
Cervix only	Any genital	4	0		61	–		6.6 (1.8–14.5)	–
Urine only	Penis	2	1		55	36		3.7 (0.4–10.2)	2.8 (0.1–10.4)
Urine only	Scrotum	3	2		55	36		3.7 (.04–10.2)	5.6 (0.7–15.7)
Urine only	Any genital	5	3		55	36		9.2 (3.0–18.8)	8.4 (1.7–20.3)
Cervix/urine only	Penis	6	2		115	83		5.2 (1.9–10.1)	2.4 (0.3–6.7)
Cervix/urine only	Scrotum	5	3		115	83		4.3 (1.4–8.9)	3.6 (0.7–8.7)
Any cervix/urine	Penis	20	12		115	83		17.4 (10.6–25.8)	14.6 (7.5–23.9)
Any cervix/urine	Scrotum	12	8		115	83		10.4 (5.4–17.1)	9.7 (4.2–17.4)
Any cervix/urine	Any genital	32	20		115	83		27.8 (19.0–38.3)	24.2 (14.8–35.9)
Any cervix/urine	Anus	1	1		115	83		0.9 (0.02–3.2)	1.2 (0.03–4.50
Any cervix/urine	Hands	1	1		115	83		0.9 (0.02–3.2)	1.2 (0.03–4.5)
Anus only	Scrotum	3	1		51	36		5.9 (1.2–14.2)	2.7 (0.1–10.1)
Any anus	Any genital	24	16		51	36		47.1 (30.2–67.7)	44.0 (25.1–68.0)
Any hands	Any genital	4	2		426	373		28.2 (7.7–61.8)	16.1 (1.9–44.8)
Self-inoculation									
Male§									
Penis only	Scrotum	5	1		102	41		4.9 (1.6–10.0)	2.4 (0.1–9.0)
Scrotum only	Penis	3	2		50	27		6.0 (1.2–14.5)	7.5 (0.9–20.9)
Any genital	Any genital	15	6		111	51		13.6 (7.6–21.3)	11.7 (4.3–22.8)
Any genital	Hands	3	2		111	51		2.7 (0.6–6.5)	3.9 (0.5–10.9)
Anus only	Any genital	3	2		29	21		10.2 (2.1–24.6)	9.3 (1.1–26.0)
Female									
Cervix/urine only	Anus	1	1		115	83		0.9 (0.02–3.2)	1.2 (0.03–4.5)
Cervix/urine/anus	Hand	3	2		166	119		1.8 (0.4–4.4)	1.7 (0.2–4.7)

*HPV, human papillomavirus; CI, confidence interval. †Based on the duration of infection in the source site. When HPV was detected at a given visit followed by the absence of that HPV type at the successive visit, the exposure period was estimated at half of the visit interval. ‡Includes probable oncogenic types. §Includes penis-to-penis subsite inoculations.

### Behavioral Factors

All couples with genital-to-genital transmission reported vaginal intercourse during the period corresponding to the transmission event. Among the 5 couples with penis-to-anus transmission, 4 reported anal intercourse during the corresponding time period.

Table 3 compares baseline characteristics of couples with and without HPV transmission. Male partners in transmitting couples had had more sexual partners over their lifetime. Transmitting couples had more frequent sexual intercourse with one another, were more likely to have contact between the male’s mouth and the female’s anus, were more likely to use birth control injections and have withdrawal before ejaculation, and had fewer periods of abstinence. Over half of nontransmitting couples reported use of condoms 100% of the time during sexual intercourse within the previous 4 months, compared with only 3% of transmitting couples.

**Table 3:** Characteristics of male-female couples by HPV transmission status*

Characteristic	Transmission (n = 16 couples)	No transmission (n = 9 couples)	p value
Mean age (SD)			
M	25.4 (8.8)	32.2 (14.4)	0.22
F	25.5 (10.2)	26.3 (7.3)	0.82
Length of relationship, mo, median (range)	10.7 (1.9–73.4)	13.1 (0.23–185)	0.22
Monthly frequency of sexual intercourse, mean (SD)	17.7 (10.0)	6.9 (7.8)	0.0002
Lifetime no. sexual partners, mean (SD)			
M	13.9 (10.4)	5.9 (5.1)	0.04
F	6.7 (6.1)	2.8 (2.2)	0.08
Circumcised man, no. (%)	13 (81)	7 (78)	0.84
Sexual practices (ever/never), no. (%)			
Vaginal intercourse	32 (100)	18 (100)	1.00
Anal intercourse	14 (44)	6 (33)	0.47
Oral-vaginal	29 (91)	13 (72)	0.09
Oral-penile	32 (100)	17 (94)	0.18
Oral (M)–anal (F)	6 (19)	0	0.05
Oral (F)–anal (M)	2 (6)	0	0.28
Finger/other object-vaginal	28 (88)	18 (100)	0.12
Finger/other object (M)–anal (F)	9 (28)	4 (22)	0.65
Finger/other object (F)–anal (M)	12 (38)	4 (22)	0.27
Contraception ever used with partner, no. (%)			
Birth control pill	21 (66)	8 (44)	0.15
Birth control shot	6 (19)	0	0.05
“Morning after” pill	15 (16)	0	0.08
Spermicides	3 (9)	2 (11)	0.84
Withdrawal	19 (59)	5 (28)	0.03
Vasectomy	2 (6)	4 (22)	0.10
Female condom	2 (6)	3 (17)	0.24
Abstinence	5 (16)	8 (44)	0.03
Condom	26 (81)	16 (89)	0.48
Frequency of condom use (prior 4 mo), no. (%)			
Always	1 (3)	10 (56)	
Never/some use†	31 (97)	8 (44)	<0.0001
Sexually transmitted infection history, no. (%)			
Chlamydia	4 (13)	0	0.12
Genital herpes	1 (3)	2 (11)	0.25
Cigarette smoking (ever)‡, no. (%)			
M	9 (56)	3 (33)	0.27
F	4 (25)	0	0.10

*HPV, human papillomavirus; SD, standard deviation. . †Some use defined as usage less than half the time, half the time, or more than half the time. ‡History of smoking daily for >6 months.

## Discussion

This study demonstrates that HPV is efficiently transmitted between sexual partners and that multiple transmission events can occur within a couple. The rates of genital transmission from women to men were substantially higher than from men to women. Greater rates of female-to-male transmission should imply higher HPV prevalence in men. Studies in men to date, including our own cohort, have reported male genital HPV prevalences at least as high as in women, with most reporting prevalences of at least 20% and up to 73% (*9*,*19*). The penis shaft was the primary source of transmission to the cervix; the cervix and urine were the primary sources of infection to male genitals.

Sexual transmission also involved the scrotum, the anus of women, and the hands of both sexes. The oral cavity and semen were not involved in transmission.

The anus of women was both a major source and target of heterosexual transmission. We observed consistency between penis-to-female anus transmission and reported anal intercourse during the corresponding period. We previously demonstrated high genotypic concordance between concurrent cervical-anal infections in women, which indicates possible common sources of infection (*20*).

Transmission through nonpenetrative sexual contact was demonstrated between the female anus and the scrotum, as well as the female hand and male genitals. The male anus was not a major source or a target of HPV transmission. However, 3 of the 4 couples with baseline infection in only 1 partner involved anal infection in men.

Male self-transmission frequently involved the scrotum, likely facilitated by passive contact between proximate genital sites. The scrotum may be an important reservoir of infection for penile infections that can subsequently be transmitted to partners. Hands may also serve as reservoirs of infection in both men and women. Auto-inoculation involving the hands may result from casual contact or masturbation.

To some extent, our study results suggest that HPV is relatively indiscriminate in its patterns of transmission. We observed the transmission of a given viral genotype to multiple anatomic sites in a partner and concurrent transmission of multiple genotypes to the same site.

Other observations suggest that HPV transmission is not entirely arbitrary and may reflect tissue or genotype differences or both. Rates of transmission of oncogenic types to the male genitals from the urine were higher than from the cervix. This may reflect differences in genotypes found in the vagina and vulva compared with those found in the cervix. All transmission events requiring extended periods of exposure involved the female anus target site.

A total of 15 genotypes were transmitted, including 13 which were transmitted through heterosexual means. HPV 16 and 18 and nononcogenic HPV 6 and 11, the 4 types included in the current quadrivalent vaccine, comprised <10% of transmitted types. Notably, we observed greater transmission of HPV 16 to the cervix than such transmission by other types, which underscores the possibility of selective transmission of some HPV types.

Compared with couples not experiencing HPV transmission, transmitting couples were more sexually active and were more likely to use certain nonbarrier forms of contraception. Few HPV-transmitting couples reported always using condoms during recent sexual activity, compared with over half of nontransmitting couples.

A major limitation of our study was the potential for misclassification of HPV transmission events. Variable detection of HPV could be due to natural fluctuation in viral levels or variable sampling of sites could confound the observation of viral transmission. For example, instances of apparent reinfection of sites may alternatively represent possible reactivation of latent infections.

Another potential source of misclassification was the lack of a priori knowledge of the time required for HPV to be acquired from an infected partner. Viral transmission could have occurred more frequently than the 2-month visit intervals used in the study, and transmission events could have been missed.

Another limitation of the study was the inclusion of couples who had already had sexual contact with one another; initial viral transmission was likely to have occurred before study entry. Indeed, nearly half of the couples had type-specific concordant infections at study entry, indicating previous transmission of HPV, which limited our ability to evaluate incident infections.

Because our study relied on self-reported sexual activity, it was subject to recall bias. Furthermore, although all persons reported monogamous relationships, some of the incident infections without a source, most of which involved the male genitals, could have been acquired through sexual activity with another partner. Despite these limitations, the present study included intensive follow-up of a well-characterized cohort, sampling of multiple genital and nongenital sites, and state-of-the-art HPV testing and genotyping methods.

The development of comprehensive HPV prevention and control strategies, which incorporate HPV vaccine usage and contraceptive practices, is impeded by lack of information on the risk and routes of sexual transmission between heterosexual partners and potential genotype-specific differences in transmission efficiency. The small size of the cohort and the diversity of genotypes precluded type-specific analysis of transmission.

This study contributes to a growing body of knowledge of HPV in men because we directly examined HPV transmission. However, study results are preliminary and need to be verified in larger cohorts. Future HPV transmission studies are critical to address major gaps in our knowledge of the natural history of this virus.

## Supplementary Material

Appendix Table HPV transmission events in male-female couples by anatomic site*

## References

[1] Schiffman MH. Recent progress in defining the epidemiology of human papillomavirus infection and cervical neoplasia. J Natl Cancer Inst. 1992;84:394–8. 10.1093/jnci/84.6.394 1311392

[2] Bosch FX, de Sanjosé S. Chapter 1: human papillomavirus and cervical cancer—burden and assessment of causality. J Natl Cancer Inst Monogr. 2003;31:3–13. 12807939 10.1093/oxfordjournals.jncimonographs.a003479

[3] Ho L, Tay SK, Chan SY, Bernard HU. Sequence variants of human papillomavirus type 16 from couples suggest sexual transmission with low infectivity and polyclonality in genital neoplasia. J Infect Dis. 1993;168:803–9. 8397266 10.1093/infdis/168.4.803

[4] Hippelainen MI, Yliskoski M, Syrjanen S, Saastamoinen J, Hippelainen M, Saarikoski S, Low concordance of genital human papillomavirus (HPV) lesions and viral types in HPV-infected women and their male sexual partners. Sex Transm Dis. 1994;21:76–82. 9071416 10.1097/00007435-199403000-00004

[5] Kyo S, Inoue M, Koyama M, Fujita M, Tanizawa O, Hakura A. Detection of high-risk human papillomavirus in the cervix and semen of sex partners. J Infect Dis. 1994;170:682–5. 8077728 10.1093/infdis/170.3.682

[6] Strand A, Rylander E, Wilander E, Zehbe I. HPV infection in male partners of women with squamous intraepithelial neoplasia and/or high-risk HPV. Acta Derm Venereol. 1995;75:312–6. 8578958 10.2340/0001555575312316

[7] Baken LA, Koutsky LA, Kuypers J, Kosorok MR, Lee SK, Kiviat NB, Genital human papillomavirus infection among male and female sex partners: prevalence and type-specific concordance. J Infect Dis. 1995;171:429–32. 7844382 10.1093/infdis/171.2.429

[8] Weaver BA, Feng Q, Holmes KK, Kiviat N, Lee SK, Meyer C, Evaluation of genital sites and sampling techniques for detection of human papillomavirus DNA in men. J Infect Dis. 2004;189:677–85. 10.1086/381395 14767822

[9] Hernandez BY, McDuffie K, Goodman MT, Wilkens LR, Thompson P, Zhu X, Comparison of physician- and self-collected genital specimens for detection of human papillomavirus in men. J Clin Microbiol. 2006;44:513–7. 10.1128/JCM.44.2.513-517.2006 16455906 PMC1392697

[10] Gravitt PE, Peyton CL, Alessi TQ, Wheeler CM, Coutlee F, Hildesheim A, Improved amplification of genital human papillomaviruses. J Clin Microbiol. 2000;38:357–61. 10618116 10.1128/jcm.38.1.357-361.2000 PMC88724

[11] Gravitt PE, Peyton CL, Apple RJ, Wheeler CM. Genotyping of 27 human papillomavirus types by using L1 consensus PCR products by a single-hybridization, reverse line blot detection method. J Clin Microbiol. 1998;36:3020–7. 9738060 10.1128/jcm.36.10.3020-3027.1998 PMC105104

[12] de Villiers EM, Fauquet C, Broker TR, Bernard HU, zur Hausen H. Classification of papillomaviruses. Virology. 2004;324:17–27. 10.1016/j.virol.2004.03.033 15183049

[13] Munöz N, Castellságue X, de González AB, Gissmann LBB. Chapter 1: HPV in the etiology of human cancer. Vaccine. 2006;24S3:S1–S10. 10.1016/j.vaccine.2006.05.115 16949995

[14] Coutlee F, Rouleau D, Petignat P, Ghattas G, Kornegay JR, Schlag P, Enhanced detection and typing of human papillomavirus (HPV) DNA in anogenital samples with PGMY primers and the linear array HPV genotyping test. J Clin Microbiol. 2006;44:1998–2006. 10.1128/JCM.00104-06 16757590 PMC1489445

[15] van Hamont D, van Ham MA, Bakkers JM, Massuger LF, Melchers WJ. Evaluation of the SPF10-INNO LiPA human papillomavirus (HPV) genotyping test and the Roche linear array HPV genotyping test. J Clin Microbiol. 2006;44:3122–9. 10.1128/JCM.00517-06 16954236 PMC1594724

[16] Giuliani L, Coletti A, Syrjänen K, Favalli C, Ciotti M. Comparison of DNA sequencing and Roche linear array in human papillomavirus (HPV) genotyping. Anticancer Res. 2006;26(5B):3939–41. 17094425

[17] Daponte A, Pournaras S, Mademtzis I, Hadjichristodoulou C, Kostopoulou E, Maniatis AN, Evaluation of high-risk human papillomavirus types PCR detection in paired urine and cervical samples of women with abnormal cytology. J Clin Virol. 2006;36:189–93. 10.1016/j.jcv.2006.03.009 16690350

[18] Dobson AJ, Kuulasmaa K, Eberle E, Scherer J. Confidence intervals for weighted sums of Poisson parameters. Stat Med. 1991;10:457–62. 10.1002/sim.4780100317 2028128

[19] Dunne EF, Nielson CM, Stone KM, Markowitz LE, Giuliano AR. Prevalence of HPV infection among men: a systematic review of the literature. J Infect Dis. 2006;194:1044–57. 10.1086/507432 16991079

[20] Hernandez BY, McDuffie K, Zhu X, Wilkens LR, Killeen J, Kessel B, Anal human papillomavirus infection in women and its relationship with cervical infection. Cancer Epidemiol Biomarkers Prev. 2005;14:2550–6. 10.1158/1055-9965.EPI-05-0460 16284377 PMC1475824

