# Supplementary material for: Transmission of Human Papillomavirus in Heterosexual Couples
Source: Emerg Infect Dis. 2008 Jun;14(6):888–94. doi: 10.3201/eid1406.070616.2 (PMC2600292; doi:10.3201/eid1406.070616.2)
Supplement: Appendix Table — HPV transmission events in male-female couples by anatomic site* [file 07-0616_appT-s1.pdf]

Appendix Table. HPV transmission events in male-female couples by anatomic site*

Couple	Source site(s)†	Visit‡	Destination site(s)§	Visit	Transmitted HPV genotype	
					Oncogenic**	Nononcogenic††
A	Anus (F)	1, 2	Scrotum	3	39	
	Scrotum	3	Shaft	4	39	
	Scrotum	3	Glans	4	39	
	Shaft	4, 5	Anus (F)	6	39	
	Shaft	5	Glans	6	39	
	Anus (F)	2	Scrotum	3		6
B	Urine (F)	1	Scrotum	2	39	
	Foreskin, scrotum	1 (foreskin only), 2	Anus (F)	3	39	
	Cervix/urine, anus	3	Foreskin	4	39	
	Cervix/urine, anus	3	Glans	4	39	
	Cervix/urine, anus	3	Scrotum	4	39	
	Cervix/urine, anus	3	Hand (M)	4	39	
	Cervix/urine, anus	3	Anus (M)	4	39	
	Cervix/urine, anus	3	Shaft	4	39	
	Cervix/urine, anus	3	Hand (F)	4	39	
	Shaft, foreskin	1	Scrotum	2		84
	Foreskin, scrotum	1 (foreskin only), 2	Anus (F)	3		84
	Scrotum, foreskin	2	Shaft	3		84
	Scrotum, foreskin	2	Glans	3		84
	Foreskin, glans, shaft	3	Cervix/urine	4		84
	Anus (F)	3	Hand (M)	4		84
	Anus (F)	3	Scrotum	4		84
C	Urine (F)	1	Hand (F)	2		CP6108
	Urine (F)	1	Shaft	2		CP6108
	Urine (F)	1	Scrotum	2		CP6108
	Shaft, scrotum	2	Cervix	3		CP6108
D	Glans	1	Hand (F)	2	56	
	Glans, scrotum	1	Cervix	2		84
	Cervix	2	Shaft	3		84
	Cervix	2	Scrotum	3		84
	Shaft	1	Cervix/urine	2	16	
	Shaft	1	Anus (F)	2	16	
	Cervix/urine, anus	2	Scrotum	3	16	
	Cervix/urine, anus, hand	2	Scrotum	3	18	
	Scrotum	1	Shaft	2		6
	Glans, scrotum	1	Shaft	2		6
	Shaft	1	Hand (M)	2		62
E	Glans, shaft, scrotum	1	Anus (F)	2	53	
	Glans, shaft, scrotum	1	Hand (F)	2	53	
F	Foreskin, glans, shaft, scrotum, hand	1	Cervix	2		CP6108
	Urine, anus (F)	1	Hand (F)	2		42
	Urine, anus (F)	1	Glans	2		42F
	Urine, anus (F)	1	Foreskin	2		42
	Urine, anus (F)	1	Scrotum	2		42
	Urine, anus (F)	1	Glans	2	59	
	Urine, anus (F)	1	Foreskin	2	59	
G	Urine, anus (F)	1	Glans	2		54
	Urine, anus (F)	1	Shaft	2		54
	Urine, anus (F)	1	Scrotum	2		54
	Urine, anus (F)	1	Glans	2	56	
	Urine, anus (F)	1	Shaft	2	56	
	Urine, anus (F)	1	Scrotum	2	56	
	Urine (F)	1	Glans	2		62
	Urine (F)	1	Scrotum	2		62
	Shaft	1	Anus (F)	2		62
	Shaft	1	Hand (F)	2		62
H	Shaft	2	Scrotum	3		84
	Cervix/urine	2	Glans	3	59	
	Cervix/urine	2	Scrotum	3	59	
I	Hand (F)	1	Glans	2		84
	Glans	2	Scrotum	3		84
J	Anus (F)	1	Scrotum	2		55
K	Cervix	1	Glans	2		54
	Cervix	1	Shaft	2		54
	Urine (M)	1	Glans	2	51	

	Urine (M)	1	Shaft	2	51	
L	Cervix/urine, anus	1	Glans	2	31	
	Cervix/urine, anus	1	Shaft	2	31	
	Cervix/urine, anus	1	Scrotum	2	31	
	Glans, shaft	4	Scrotum	5	31	
	Shaft, scrotum	3	Glans	4		6
	Shaft	4	Scrotum	5		6
	Cervix/urine	1, 2, 3	Anus (F)	4	16	
M	Shaft, scrotum	1	Hand (M)	2	51	
	Cervix/urine, anus, hand	2	Glans	3		62
N	Anus (M)	1	Shaft	2	18	
O	Anus (M)	1	Shaft	2	18	
P	Glans, shaft, scrotum	1	Hand (M)	2	39	
